# Effects of Supervised Early Resistance Training versus standard care on cognitive recovery following cardiac surgery via median sternotomy (the SEcReT study): protocol for a randomised controlled pilot study

**DOI:** 10.1186/s13063-020-04558-x

**Published:** 2020-07-15

**Authors:** Jacqueline M. S. Pengelly, Alistair G. Royse, Adam L. Bryant, Gavin P. Williams, Lynda J. Tivendale, Timothy J. Dettmann, David J. Canty, Colin F. Royse, Doa A. El-Ansary

**Affiliations:** 1 Department of Nursing and Allied Health, Swinburne University of Technology, Hawthorn, Melbourne, Victoria Australia; 2 Department of Surgery, University of Melbourne, Melbourne, Victoria Australia; 3 Department of Cardiothoracic Surgery, Royal Melbourne Hospital, Parkville, Victoria Australia; 4 Department of Physiotherapy, University of Melbourne, Parkville, Victoria Australia; 5 Kieser Australia, South Melbourne, Victoria Australia; 6 Department of Medicine, Monash University, Clayton, Victoria Australia; 7 Department of Anaesthesia and Perioperative Medicine, Monash Health, Clayton, Victoria Australia; 8 Department of Anaesthesia and Pain Management, Royal Melbourne Hospital, Parkville, Victoria Australia; 9 Australian Director, Outcomes Research Consortium Cleveland Clinic, Cleveland, Ohio USA; 10 Clinical Research Institute, Westmead Private Hospital, Westmead, Sydney, NSW Australia

**Keywords:** Median sternotomy, Resistance training, Cognition, Cardiac surgery, Recovery, Rehabilitation, Exercise

## Abstract

**Introduction:**

Mild cognitive impairment is considered a precursor to dementia and significantly impacts upon quality of life. The prevalence of mild cognitive impairment is higher in the post-surgical cardiac population than in the general population, with older age and comorbidities further increasing the risk of cognitive decline. Exercise improves neurogenesis, synaptic plasticity and inflammatory and neurotrophic factor pathways, which may help to augment the effects of cognitive decline. However, the effects of resistance training on cognitive, functional and overall patient-reported recovery have not been investigated in the surgical cardiac population. This study aims to determine the effect of early moderate-intensity resistance training, compared to standard care, on cognitive recovery following cardiac surgery via a median sternotomy. The safety, feasibility and effect on functional recovery will also be examined.

**Methods:**

This study will be a prospective, pragmatic, pilot randomised controlled trial comparing a standard care group (low-intensity aerobic exercise) and a moderate-intensity resistance training group. Participants aged 18 years and older with coronary artery and/or valve disease requiring surgical intervention will be recruited pre-operatively and randomised 1:1 to either the resistance training or standard care group post-operatively. The primary outcome, cognitive function, will be assessed using the Alzheimer’s Disease Assessment Scale and cognitive subscale. Secondary measures include safety, feasibility, muscular strength, physical function, multiple-domain quality of recovery, dynamic balance and patient satisfaction. Assessments will be conducted at baseline (pre-operatively) and post-operatively at 2 weeks, 8 weeks, 14 weeks and 6 months.

**Discussion:**

The results of this pilot study will be used to determine the feasibility of a future large-scale randomised controlled trial that promotes the integration of early resistance training into existing aerobic-based cardiac rehabilitation programs in Australia.

**Trial registration:**

Australian New Zealand Clinical Trials Registry (ANZCTR) ACTRN12617001430325p. Registered on 9 October 2017. Universal Trial Number (UTN): U1111-1203-2131.

## Introduction

### Background and rationale

Following cardiac surgery, mild cognitive impairment (MCI) affects 15–40% of patients, with the elderly affected at a greater rate [1–3]. This has the potential to become a significant health problem, as mild cognitive impairment is considered a precursor to dementia [4, 5]. Cardiac surgery patients are at a 30% risk of progression to dementia within 7.5 years of surgery [6] compared to 2.5% in the general population [5]. Following cardiac surgery, patients often require assistance with many activities of daily living; however, the personal and emotional cost of dementia is long lasting, including a loss of independence and self-control, strain on family and carers, depression and anxiety [7]. Furthermore, the economic cost of dementia in Australia, including hospitalisations, aged care costs and pharmaceuticals, was $14.25 billion in 2016, $8.8 billion (62%) of which involved direct costs to the healthcare system [7].

The cardiac surgery population is an ageing one with a general average age of 70 years old in most western countries and most present with multiple comorbidities [8]. Exercise, inclusive of aerobic and resistance training, can help to simultaneously manage numerous comorbidities; however, current cardiac rehabilitation programs are often of a low intensity and do not take into consideration a patient’s individual comorbidities and risk factors [9]. Furthermore, despite the high prevalence of cognitive impairment, cognitive recovery is not specifically assessed or considered in current cardiac rehabilitation programs, and the focus of programs to date has been on screening for depression, physical outcomes and at times functional outcomes [10].

It has been postulated that progressive resistance training augments the effects on insulin-like growth factor-1 and insulin sensitivity and mediates inflammation and neurotrophic factor pathways associated with cognitive decline and sarcopenia [11, 12]. Sarcopenia is a factor in increased falls risk, functional decline and frailty in this population [13]. Furthermore, a recent systematic review by Pengelly et al. found that the addition of resistance training to standard care, consisting predominantly of aerobic training, may lead to greater improvements in physical and functional post-operative recovery. However, it was identified that despite this finding, further research is required to investigate the safety and efficacy of specific resistance training following median sternotomy procedures and its effect on cognitive function [10].

Resistance training exercises have been reported to require focused and targeted coordinated input from, and blood flow to, all areas of the brain, which is believed to enhance neuroplasticity and neurogenesis [14, 15] at the levels of the central nervous system [11] and neurohormonal axis [12, 16].

Moreover, a recent review found that the performance of resistance training following median sternotomy appears to be safe and feasible, and results in similar improvements in both cardiopulmonary capacity and anthropometry, when compared to aerobic training alone [17]. Therefore, resistance training may be more effective at improving cognitive recovery beyond that of aerobic training alone. However, there are numerous problems with the translation and application of resistance training. This includes the misclassification of callisthenic-based exercises as resistance training; the resistance training programs do not meet the American College of Sports Medicine guidelines [18], and sternal stability has not been monitored prior to commencing or progressing upper limb exercises. Consequently, we do not know the effect of specifically designed resistance training interventions on post-operative recovery (i.e. in compliance with ACSM guidelines), nor has the effect of any mode of exercise on cognitive recovery in the surgical cardiac population been investigated [10, 17].

### Objectives

This study aims to examine the effect of moderate-intensity resistance training compared to a control group who receive standard cardiac rehabilitation involving low-intensity aerobic training within the community setting on post-operative cognitive recovery following cardiac surgery via median sternotomy. The safety, feasibility, patient satisfaction and effect of the exercise interventions on overall post-operative recovery, muscular strength, balance, lung capacity, upper limb function, independence with activities of daily living and anthropometry will also be examined. As this is a pilot study, the data obtained will be used to inform a sample size calculation to be performed for a larger definitive study.

## Methods

This prospective pragmatic pilot randomised controlled trial will be conducted in accordance with the National Statement on Ethical Human Research and the Australian Code for the Responsible Conduct of Research. Ethical approval has been granted from the Human Research Ethics Committee from Melbourne Health (Application ID: 2017.266). Local governance approval will be obtained prior to commencement of trial recruitment at other centres. All participants will provide written informed consent prior to commencing the study. The study follows the CONsolidated Standards of Reporting Trials (CONSORT) 2010 guidelines. This protocol includes the items identified in the Standard Protocol Items: Recommendations for Interventional Trials (SPIRIT) 2013 checklist (Additional file 1). The trial was prospectively registered with the Australian New Zealand Clinical Trials Registry (ACTRN12617001430325p).

### Study design and setting

**Fig. 1:**
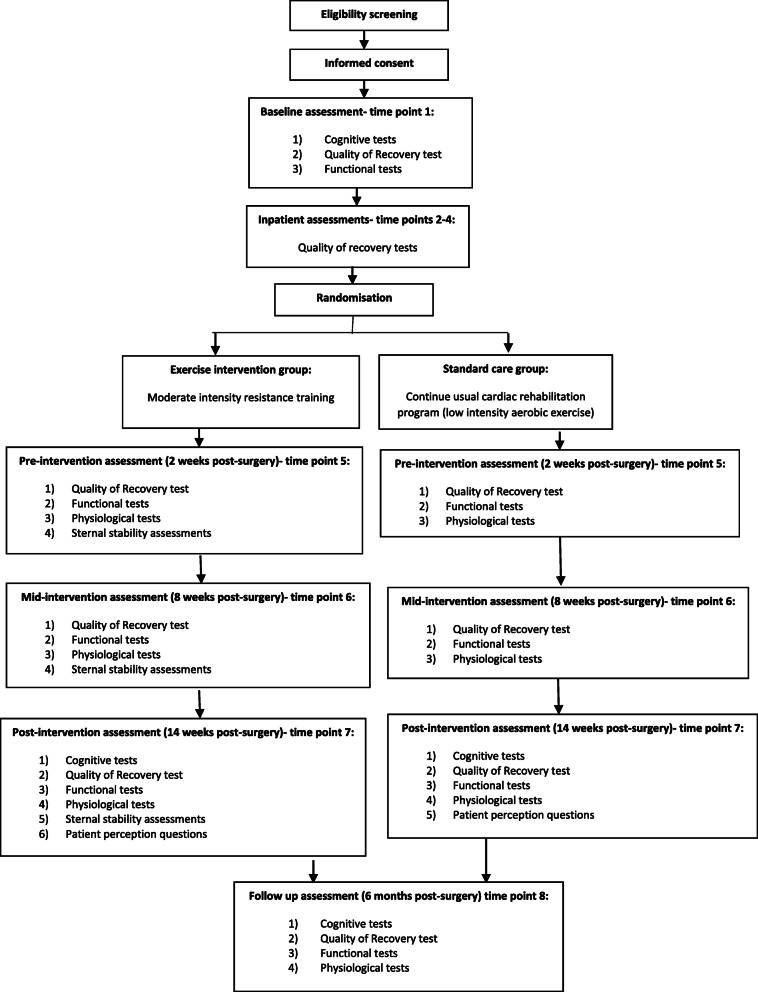
Flow diagram of study procedure, recruitments and randomisation

The SEcReT study is designed as a multi-centre prospective pragmatic pilot randomised controlled trial with two parallel groups: (i) moderate-intensity resistance training and (ii) standard care, which involves low-intensity aerobic training. The SEcReT study will be conducted in Melbourne, Australia. Baseline measures will be obtained at T0 (pre-surgery) to allow comparison with post-operative outcomes (Fig. 1). The groups will then be assessed at T1 (day 1), T2 (day 3), T3 (day 6), T4 (2 weeks post-operatively/pre-intervention), T5 (8 weeks post-operatively/mid-intervention), T6 (14 weeks post-operatively/post-intervention) and T7 (6 months post-intervention) to examine the effect of the intervention on cognitive, physical and functional recovery.

### Participants and recruitment

Participants will be recruited from the Royal Melbourne and Melbourne Private hospitals. Theatre and pre-admission clinic lists will be screened for patients undergoing cardiac surgery procedures via median sternotomy. Patients will then be approached about participating in the study and written informed consent provided prior to commencing data collection. Participants will be eligible if they meet the following inclusion criteria: (1) aged 18 years and older; (2) undergoing elective cardiac surgery via a median sternotomy, inclusive of coronary revascularisation and/or valve surgery; and (3) sufficient English to complete the questionnaires. If assistance to complete the questionnaires is required, the patient will be excluded from the study.

Participants with a dementia diagnosis, a musculoskeletal condition limiting ability to exercise or a geographical residence and/or lack of access to transportation, thus preventing exercise session attendance, will be excluded from this study. Furthermore, patients undergoing revision procedures that include a re-sternotomy will be excluded, as revision surgeries have been found to increase the risk of sternal complications, such as sternal infection and delay sternal healing [19]. There may also be an additional impact on sternal biomechanics, due to changes in sternal incision and bone integrity; however, this has not been investigated. Prior to commencement of data collection, written informed consent will be obtained from each participant.

### Randomisation, allocation concealment and blinding

All participants will be randomly allocated (1:1) to either the moderate-intensity resistance training group or the control group (standard care). Randomisation will be undertaken in blocks of 4–8, using double opaque envelopes. Envelopes will be prepared by an independent person not involved in the study. Patients will be randomised prior to discharge from hospital, following baseline data collection. The assessor is responsible for delivering the resistance training intervention, in addition to outcome measure assessment at all time points; therefore, she will not be blinded to group allocation. It will also not be possible to blind participants to their respective group allocation.

### Sample size determination

As this is a pilot study, a sample size calculation to determine the power of this study will not be performed. Instead, the focus will be on the feasibility and safety of the study. We plan to recruit a minimum of 20 participants, with an 80% retention rate.

### Procedure and data collection

The procedure is outlined in Fig. 1. Initial screening will be conducted by one of the researchers (JP) based on weekly theatre lists and pre-admission clinic lists. Patients who meet the inclusion criteria will have their medical histories screened to ensure suitability and eligibility. Patients who satisfy all criteria will be approached for participation in person by one of the researchers (JP), either in the pre-admission clinic or upon admission to the ward prior to their scheduled surgery. All patients will undergo the following functional assessments including lung capacity, body mass index, waist circumference, muscular strength, sternal stability and dynamic balance (Table 1). Measures of post-operative quality of recovery, cognition and upper limb function will also be recorded through questionnaires. Patient’s satisfaction with the exercise program and their perception of benefits and barriers will be assessed at the T6 (post-intervention) data collection time point.

**Table 1:** Domains and outcome measures

Domain	Outcome measure	Time point for data collection
Safety	Major adverse cardiac and cerebral events	6 months
Cognition	Alzheimer’s Disease Assessment Scale and cognitive subscale [20, 21] Mini Mental State Examination [22, 23]	Baseline, 14 weeks, 6 months
Quality of recovery	Postoperative Quality of Recovery Scale [24]	Baseline, 1 day, 3 days, 6 days, 2 weeks, 8 weeks, 14 weeks and 6 months
Cardiovascular	Lung capacity [25] Heart rate Oxygen saturation Blood pressure	Baseline, 2 weeks, 8 weeks, 14 weeks and 6 months
Physical health	Weight Waist circumference Body mass index	Baseline, 2 weeks, 8 weeks, 14 weeks and 6 months
Strength	Isometric knee flexion strength Isometric knee extension strength Hand grip strength [26]	2 weeks, 8 weeks, 14 weeks and 6 months
Balance	Four square step test [27, 28]	2 weeks, 8 weeks, 14 weeks and 6 months
Independence	Instrumental Activities of Daily Living scale [29]	Baseline, 14 weeks and 6 months
Upper limb impairment	Functional Difficulties Questionnaire [30]	Baseline, 2 weeks, 8 weeks, 14 weeks and 6 months
Sternal stability	Sternal ultrasound [31] Sternal Instability Scale [34]	2 weeks, 8 weeks, 14 weeks and 6 months
Participant satisfaction	Patient perception questions [35]	14 weeks

The study schedule is outlined in Table 2, in accordance with the Standard Protocol Items: Recommendations for Interventional Trials (SPIRIT) figure. Participants will attend either The Royal Melbourne Hospital or Melbourne Private Hospital for baseline testing. This will include attainment of all outcome measures, excluding muscular strength, sternal stability and dynamic balance. During their hospital admission, quality of recovery will be assessed on the ward at T1, T2 and T3. Patients will then be randomised to either the (1) moderate-intensity resistance training intervention or (2) standard care group (control group). Strength and balance testing will be performed during the first post-operative visit, prior to commencing the allocated exercise intervention. This will occur approximately 2 weeks post-operatively. Patients in the resistance training group will attend Kieser Essendon, Caulfield or South Melbourne twice a week for 12 weeks (total of 24 exercise sessions). Patients in the control group will be referred to cardiac rehabilitation by their respective hospital, as per standard post-operative care procedures. All participants will be re-assessed at T5 (mid-intervention), T6 (post-intervention) and T7 (6 months follow-up). Patients will be contacted a week prior to their scheduled appointment to confirm attendance. Patients will also be told that they can contact the student researcher at any time during the study if they have any questions or concerns about their recovery. Cognitive assessments will be undertaken at T0 (baseline), T6 (post-intervention) and T7 (6 months follow-up).

**Table 2:**
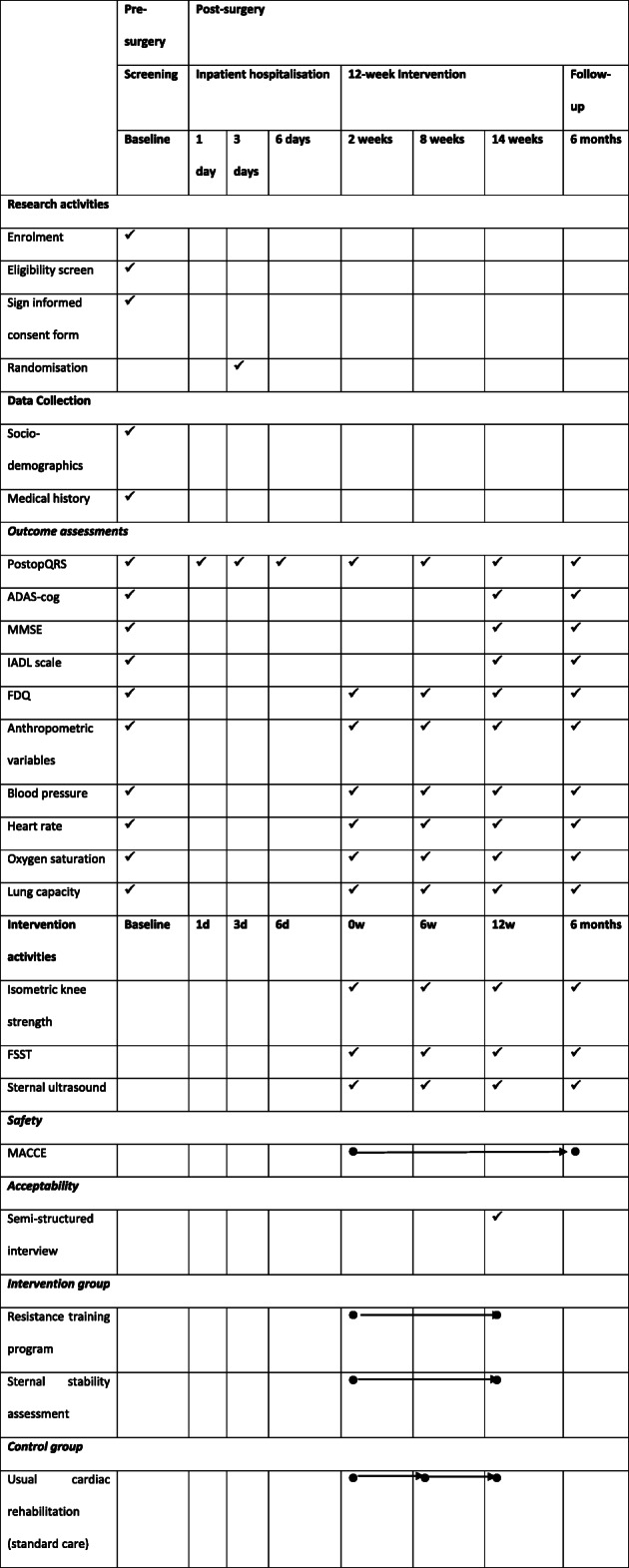
Study design schedule in accordance with the Standard Protocol Items: Recommendations for interventional Trials (SPIRIT). *MACCE* major adverse cardiac and cerebral events, *ADAS-cog* Alzheimer’s Disease Assessment Scale and cognitive subscale, *MMSE* Mini Mental State Examination, *IADL* Instrumental Activities of Daily Living, *PostopQRS* Postoperative Quality of Recovery Scale, *FDQ* Functional Difficulties Questionnaire, *FSST* Four Square Step Test

#### Withdrawals and replacements

Participants will be informed that they are free to withdraw from the study at any time. They will also be informed that their withdrawal will not affect the relationship between them and the treating doctors or the hospitals. They will be informed that any data collected to point of withdrawal will be used to prevent bias in the results, unless they specifically refuse that request. From the time of withdrawal, no further data will be acquired for that participant.

Participants will not be replaced if they withdraw from the study. Data collected to point of withdrawal will be used to prevent bias in the results. As this is a pilot study, this data is required to determine study feasibility and group separation and will represent a low risk of bias to the study.

### Exercise intervention

#### Moderate-intensity resistance training group

Participants randomised to the moderate-intensity resistance training group will receive an exercise program to be performed twice a week for 12 weeks. The program will initially include 6 exercises targeting the lower limbs, upper limbs, neck and back, with an additional exercise added after the first week. Following the T6 (mid-intervention) assessment, patients will progress to a program consisting of 11 exercises (see Table 3). The exercise program will include the following exercises: leg press, knee extension, knee flexion, hip abduction, hip adduction, latissimus pulldown, shoulder press, seated row, triceps extension, biceps curls, lateral raise, lumbar extension and neck (cervical) extension. The exercise sessions will be delivered by a qualified exercise physiologist. Participants will be instructed to perform the exercises at a 4-2-4 tempo; that is, 4 s concentric contraction, 2 s isometric hold, 4 s eccentric contraction. Participants will be instructed to complete a single set until volitional fatigue or will be instructed to stop by the exercise physiologist when technique falters. When working at a moderate intensity, each exercise will be performed for a period of 90–120 s, or 9–12 repetitions.

**Table 3:** Details of the resistance exercises and exercise progression

	Exercise	Target muscle group(s)	Sets x Reps	Intensity	Progression
**Weeks 1–6**					
1	Knee extension*	Quadriceps	1 × 9–12	RPE 13–16/20	Progressed by 5–10% of the initial weight until patients are able to exercise within the desired range (RPE 13/20 or volitional fatigue after 9–12 repetitions)
2	Knee flexion*	Hamstrings			
3	Bicep curl	Biceps			
4	Lateral raise	Deltoids			
5	Triceps pushdown	Triceps			
6	Back extension**	Lumbar extensors			
7	Neck extension	Cervical extensors			
**Weeks 7–12**					
1	Leg Press	Gluteals, hamstrings and quadriceps	1 × 9–12	RPE 13–16/20	Progressed by 5–10% of the initial weight until patients are able to exercise within the desired range (RPE 13/20 or volitional fatigue after 9–12 repetitions)
2	Hip abduction	Hip abductors			
3	Hip adduction	Hip adductors			
4	Latissimus pulldown	Latissimus dorsi			
5	Shoulder press	Deltoids and trapezius			
6	Seated row	Latissimus dorsi, trapezius, deltoids			
7	Bicep curl	Biceps			
8	Lateral raise	Deltoids			
9	Triceps pushdown	Triceps			
10	Back extension**	Lumbar extensors			
11	Neck extension	Cervical extensors			

*Progressed to Leg Press in weeks 7–12, **progressed from 0–45° to 0–55° trunk flexion in weeks 7–12

*RPE* Rating of perceived exertion

##### Exercise protocol

Measures of sternal micromotion of the sternal edges will be acquired by ultrasound imaging during each upper limb exercise with a 20-lb. resistance (the lightest weight), prior to commencing the resistance training intervention session. Sternal stability will also be assessed prior to the exercise intervention by physical examination and using the Sternal Instability Scale (SIS) [31]. If an increase in sternal micromotion > 2 mm is detected from rest and coughing, upper limb resistance training will not be commenced, or progressed, if sternal micromotion is detected with an increase in resistance. If no increase in sternal micromotion is detected, patients will continue with the initial starting weight of 20 lb. until they are familiar with the exercise equipment and technique. Participants will perform each exercise until volitional muscle fatigue, completing between 9 and 12 repetitions or achieving a rating of perceived exertion (RPE) of 13–16/20 on the Borg scale [36].

##### Exercise progression protocol

The 12-week program will be divided into two phases. For the first week, participants will commence with 6 exercises. From weeks 2–6, an additional exercise will be added (Additional file 2). After the T5 (mid-intervention) assessment, exercises will be progressed from open kinetic chain exercises using isolated single muscle movements to large compound movements using multiple muscle groups. The final 6 weeks of the program will consist of 11 exercises, each performed until volitional muscle fatigue and progressed according to the aforementioned progression protocol (Additional file 3). Additional weight will be progressively added in increments of 5–10% of the initial weight (1–2 lb. for upper limb exercises, 4–10 lb. for lower limb exercises) until patients are able to exercise within the desired range (RPE 13/20 or volitional fatigue after 9–12 repetitions).

##### Pain-level documentation and cessation of exercise rules

Pain will be defined as an increase in pain from the initial pain rating as a direct result of an exercise and assessed using the Numeric Rating Scale (0–10). An exercise will be immediately ceased with reports of sternal pain or discomfort, any sharp increase in pain or adverse event.

##### Adverse events

Adverse events will be classified as adverse events (AEs) or serious adverse events (SAEs). An AE will be defined as an incident that requires the cessation of exercise for safety reasons and is likely to be directly related to the exercise program. If an adverse event is life-threatening, requires hospitalisation and results in significantly reduced capacity or disability, or death, it will be classified as a SAE. This will be inclusive of new or unrelenting chest pain, any report of shortness of breath that does not settle quickly with rest, or acute changes in level of consciousness during the session will be considered as serious adverse events. Any serious adverse event will be immediately reported to the Ethics Committee. If there is an unacceptable risk of serious adverse events in one or both of the treatment groups, the trial will be stopped and termination may be recommended by investigators.

#### Standard care group

Standard care typically involves attending a cardiac rehabilitation program once a week for 6 weeks that is predominantly aerobic in nature and often features a progressive walking program and gentle range of motion exercises [9, 37]. Upper limb resistance is limited to a combined weight of < 1 kg for the first 8–12 post-operative weeks, or until the sternum unites, as per current sternal precautions. During the 12 weeks, participants in the standard care group will be referred by their respective hospital, to a hospital or community-based cardiac rehabilitation program, as per the hospital’s current cardiac rehabilitation referral procedures.

### Outcome measures

#### Primary outcome

##### Cognitive function

Improvement in cognitive function, particularly executive functioning, has been associated with exercise [11, 38–41]. Executive functioning refers to the cognitive processes, including working memory, multitasking, scheduling and planning [42]. One of the assessment instruments used to assess cognition will be the Alzheimer’s Disease Assessment Scale and cognitive subscale (ADAS-cog). The ADAS-cog is a tool that measures impairment across several cognitive domains, including language, orientation, constructional praxis, ideational praxis and memory [20]. It is sensitive to changes in cognitive impairment and progression to dementia [43]. Cognitive function using the ADAS-cog will be assessed at baseline (pre-operatively), 14 weeks post-operatively and 6 months post-operatively.

#### Secondary outcomes

##### Safety and feasibility

Recruitment rate and retention rate will be analysed to determine study feasibility. Feasibility data will be analysed as follows: recruitment rates and patient recruitment-to-screening ratio, percentage of participants who complete the intervention, overall percentage of sessions attended and number of participants who drop out of the study. Data for safety will be analysed as the number of sessions and exercises stopped due to pain, any adverse event or incident that stops the participant from completing the prescribed exercise, and any major adverse cardiac and cerebral events (MACCE). MACCE endpoints include all-cause mortality, cerebral vascular event (stroke), documented myocardial infarction or repeat coronary intervention (percutaneous coronary intervention or redo cardiac surgery), with data being collected at the 6 months.

##### Demographics

Demographic information including age, employment, education, smoking history, alcohol consumption, comorbidities, waist circumference and body mass index (BMI) will be collected at baseline.

##### Cognitive test

The Mini Mental State Examination (MMSE) is a valid tool commonly used to assess cognitive function. Mild cognitive impairment is a clinical diagnosis and is a recognised precursor to the development of dementia. Formal diagnosis involves assessment of cognition, especially of memory, and using an instrumental activities of daily living scale (such as the Lawton and Brody Instrumental Activities of Daily Living (IADL) scale) [29], with exclusion if there is a physical explanation for failure of IADL. There are multiple approaches used by clinicians to assess memory, but the MMSE is a commonly used tool. The MMSE was selected, as it separates patients with cognitive disturbances from those without, indicating level of cognitive impairment [22]. For the purposes of this trial, pre-existing cognitive impairment will be defined as:

- Normal—MMSE scores 27–30 and normal IADL (score 8 females and 5 males)
- Mild—MMSE scores 24–26 and normal IADL (score 8 females and 5 males)
- Moderate—MMSE scores 18–23 and normal IADL (score 8 females and 5 males)
- Severe—MMSE score < 18 and impaired IADL (score < 8 females and < 5 males)

The ADAS-cog may be susceptible to floor and ceiling effects; therefore, the Mini Mental State Examination and cognitive domain of the Postoperative Quality of Recovery Scale (PostopQRS) will also be used to ascertain changes in cognition from baseline, across time points, as well as the MMSE being used to determine baseline cognitive level. This will be assessed at baseline (pre-operatively), 14 weeks post-operatively and 6 months post-operatively.

##### Quality of recovery

Quality of recovery will be measured at all data collection time points using the Postoperative Quality of Recovery Scale (PostopQRS), which is a multidimensional survey tool that assesses quality of recovery following surgery and anaesthesia [24]. The PostopQRS assesses quality of recovery over five domains including physiologic, nociceptive, functional, cognitive and emotional recovery, as well as overall patient perspective. Scoring recovery involves comparing post-surgery performance to pre-surgery baseline values. The pre-surgery baseline values serve as an excellent phenotype for patients with respect to the five domains. The scale is conducted either face-to-face whilst in hospital or via telephone interview after discharge [44]. The ability to conduct the scale via telephone makes this a highly feasible tool for extended patient follow-up. Importantly, the scale only takes 5–6 min to conduct on each occasion and does not require highly specialised staff to perform it.

Specifically, the cognitive domain examines both memory and executive function (word generation task), allowing more than just memory to be tested [45]. Because the scale has both cognitive and functional dimensions (assessment of activities of daily living), it is suitable to use as an early indicator screening tool for cognitive impairment and dementia, as cognitive impairment can be identified with or without impairment of function. Further, the scale has defined cutoff scores for low baseline cognition which can identify preoperative cognitive impairment [44]. The application of the scale is appropriate for patients having hospital admission, as it will be used to track change over time from their baseline values on admission. It is already validated and used widely and is set up for multi-user web-based access (www.postopQRS.com). Scoring of the scale is automatically conducted by the system, and individual recovery outcomes are available in real time.

Pre-operatively, the PostopQRS can be used to capture the multidimensional aspects of quality of recovery from the patient perspective. This can be used to identify the association of cognitive decline other quality of recovery parameters. Because it is a simple to perform tool that is validated for telephone use, the PostopQRS can be used to track deterioration in cognitive and functional domains over time and will complement the assessment using the ADAS-cog and MMSE scales. This will be assessed at every time point.

##### Functional tests

1. Activities of daily living will be assessed using the Brody and Lawton Instrumental Activities of Daily Living (IADL) instrument [29]. The Lawton and Brody IADL Scale assesses the living skills deemed necessary to live independently in the community. These skills are considered more complex than the basic activities of daily living. It is useful to determine how an individual is currently functioning and monitoring improvement or deterioration over time [29]. This test has been associated with cognitive impairment and is used in conjunction with cognitive assessments to diagnose dementia and the level of cognitive impairment; however, it has not been validated specifically in cardiac patients [29, 46]. This will be assessed at baseline (pre-operatively), 14 weeks post-operatively and 6 months post-operatively.
2. Upper limb function will be assessed using the short-form Functional Difficulties Questionnaire (FDQs) [30]. The FDQs is a 10-item questionnaire which asks the participant to rate the difficulty they experience when completing a series of 10 upper limb and trunk activities by placing a mark along a 10-cm line, with anchors indicating “no difficulty” and “maximum difficulty” [30]. For those activities that participants could not attempt whilst completing the questionnaire due to the institution sternal precautions, they are asked to think back to the last time they performed the task [30]. Individual scores, measured to the nearest centimetre (cm), are aggregated to form a total out of 100 with higher scores representing greater difficulty experienced during functional activities. The FDQs has been reported to be reliable and valid in a cohort of cardiac surgery patients [47]. This will be assessed at baseline (pre-operatively); 2, 8 and 14 weeks post-operatively; and 6 months post-operatively.
3. Lung capacity will be assessed through breath gas analysis [25], using a Spirobank II Advanced Spirometer (Medical International Research, Sequim, USA) to determine forced vital capacity (FVC) and forced expiratory volume in 1 s (FEV1). Each test takes no longer than 30 s to complete. The participant will be asked to breathe normally for 3 breath cycles before being asked to take a deep breath in, breathe out as hard and as fast as they can before taking another deep breath in. This will be repeated three times with the best of the three attempts recorded. All volumes will be recorded in litres. This will be assessed at baseline (pre-operatively); 2, 8 and 14 weeks post-operatively; and 6 months post-operatively.
4. Leg strength will be assessed using isometric quadriceps and hamstring strength tests on resistance machines that stabilise the joint and isolate the muscle during testing. Each test is of 15–20-s duration to ensure safety. Peak torque is measured in Newtons, using a dynamometer placed in the shaft of the equipment. The tests will be attempted three times with the highest strength measurement recorded and noted in comparison to the age and gender normative values. This will be assessed at 2, 8 and 14 weeks post-operatively and 6 months post-operatively.
5. Handgrip strength will be assessed using a JAMAR hand-held dynamometer and measured in kilogrammes (Sammons Preston Rolyan, Brooklyn, USA). Hand-held dynamometry is a reliable, objective tool for muscle strength measurement, and a predictor of postoperative complications, mortality, functional decline and cardiovascular risk [26]. The participant will be tested in the position recommended by the American Society for Hand Therapists (participant seated with shoulder adducted, neutrally rotated, elbow flexed to 90°). The peak value of the maximal squeeze over 5 s will be recorded. Three serial tests of maximum grip strength with the right hand will be performed, and the best of the 3 values will be recorded and noted in comparison to age and gender normative values. The test is a reliable and responsive measure for patients in cardiac rehabilitation (ICC = 0.97) [26]. This will be assessed at 2, 8 and 14 weeks post-operatively and 6 months post-operatively.
6. Dynamic balance will be assessed using the Four Square Step Test (FSST), which requires rapid dynamic weight shifting, coordination and stepping [27, 48]. Participants will be asked to step as quickly as possible into four squares marked on the floor with red sticks in a certain sequence that requires the participant to step forward, backward and sideways. The test will be attempted three times with the fastest time taken to complete the sequence recorded in seconds. The researcher will act as a spotter, standing adjacent to the participants during the performance of the test, to reduce the possible risk of falls. Mobility issues have been found to affect a third of cardiac patients in the early post-operative period, thus increasing falls risk [13]. The FSST balance performance has been found to be predictive of future falls and is able to distinguish non-fallers from single fallers from multiple fallers in community-dwelling older adults and in numerous musculoskeletal and neurological conditions; however, it has not been validated in the surgical cardiac population [27, 28]. This will be assessed at 2, 8 and 14 weeks post-operatively and 6 months post-operatively.
7. Physiological data will be obtained through measurement of blood pressure, heart rate and oxygen saturation at rest, at the baseline (pre-operatively); 2, 8 and 14 weeks post-operatively; and 6 months post-operatively. All blood pressure measurements will be taken from the left arm using an Elitecare single hand sphygmomanometer and blood pressure cuff and a dual head Liberty lightweight aluminium stethoscope. Should a patient’s blood pressure be unable to be taken due to pre-existing medical reasons (i.e. fistula), it will be taken on the right arm and documented as such.
  Heart rate and oxygen saturation will be assessed using a Heart Sure A320 Pulse Oximeter (Omron, Melbourne, AUS), a portable finger pulse oximeter.

##### Sternal stability

Sternal stability will be assessed and quantified using sternal ultrasound and the Sternal Instability Scale (SIS) [31]. This will allow the researchers to measure any sternal movement occurring and monitor patient safety prior to commencing all resistance upper limb exercise modalities.

1. Sternal ultrasound: Ultrasound (US) measures of sternal micromotion will be acquired by imaging the sternum at rest, cough and on each of the six upper limb resistance training exercises. This will be stored as a video file to allow analysis of data. Measurements will be made at two sites (i) at 6 cm (mid-sternum) and (ii) 10 cm (lower sternum) from the sternal notch (Additional file 4). The greatest degree of horizontal, vertical and diagonal sternal separation (mm) will be determined by a researcher with a minimum of 1-year experience in sternal ultrasound and DICOM analysis. The video will be watched and the greatest separation recorded for each movement, at each site from a still frame. Previous research has shown that ultrasound measures are a valid and reliable indicator of the separation and motion at the sternal edges in patients post-median sternotomy [32]. US has demonstrated excellent test-retest reliability with intra-class correlation coefficients ranging from 0.90 to 0.93 [32]. A Fujifilm Sonosite iViz and a L38v 9-cm 10–5-MHz linear transducer will be used to measure sternal micromotion (Fujifilm Sonosite, Brookvale, AUS). Images will be analysed using Prosolv5 Synapse Cardiovascular. This will be assessed at 2, 8 and 14 weeks post-operatively for the resistance training participants on the six upper limb resistance exercises and during a cough at baseline and 6 months post-operatively for both the resistance training and standard care groups.
2. Sternal Instability Scale (SIS): The SIS is a physical examination test that subjectively evaluates the stability of the sternum based on a 4-point scale (Table 4) [31]. A score of 0 corresponds to a clinically stable sternum with no detectable motion or separation of the sternal edges, whilst a score of 3 corresponds to a completely separated sternum with marked increased motion or separation of the sternal edges. Patients’ initial sternal stability will be determined during rest and special testing, including cough and trunk rotation (Table 4). Whilst performing the upper limb exercises, the exercise will not be performed or the weight progressed if any increase in sternal micromotion that exceeds the score determined during the initial rest and special testing. Previous research has shown that the SIS is both a valid and reliable clinical tool for measuring the stability of the sternum in patients following a median sternotomy. It has demonstrated excellent inter- and intra-rater reliability, with intra-class correlation coefficients of 0.97 and 0.98, respectively [49]. This will be assessed at 2, 8 and 14 weeks post-operatively for the resistance training participants on the six upper limb resistance exercises. It will also be assessed when an upper limb exercise resistance is progressed and will be documented if a result other than zero (0) is found to occur.

**Table 4:** Sternal Instability Scale

Grade of motion	Modified Sternal Instability Scale
0	Clinically stable sternum (no detectable motion)—normal
1	Minimally separated sternum (slight increase in motion upon special testing*)
2	Partially separated sternum—regional (moderate increase in motion upon special testing*)
3	Completely separated sternum—entire length (marked increase in motion upon special testing*)

*Special testing may include shoulder flexion, trunk rotation, lateral flexion, coughing and opposing movements of the upper limbs, either unilaterally or bilaterally

##### Participant satisfaction

The Global Rating of Change scale will be used to subjectively assess participant satisfaction with the exercise program at the completion of the 12-week exercise program [35]. In addition, participants will be prompted to complete eight semi-structured questions about the exercise program. Questions will explore factors impacting cardiac rehabilitation participation, including perceived facilitators, barriers, benefits to recovery and enjoyment. The responses given to these questions will undergo qualitative analysis. This will be conducted at 14 weeks post-operatively.

### Data management

Information collected from participants will be stored securely in both hard copy (paper) and soft copy (electronic). The information will be stored on paper records at the University of Melbourne in a locked cabinet, and the de-identified data entered into an electronic web-based database (www.postopQRS.com, located at City, University of London) and other research documents stored on a secure file server based at the University of Melbourne.

All database storage is protected with login and password for individual researchers. All paper records used during the study are kept after the project has been completed for a minimum of 15 years, as per Good Clinical Practice. After the 15 years, information will be disposed of via shredding of all paper records, and deletion from databanks. Re-identifiable data is used during the conduct of the study in order to contact patients for follow-up visits. The code will be kept separately to the case report forms.

### Data analysis

The objective of this pilot study is to test the feasibility of recruitment, retention, intervention and data collection, and the safety of moderate-intensity resistance training, on post-operative cognitive and functional recovery. The following parameters would indicate clinically important relative differences between groups:

1. Group separation: ≥ 20% difference between groups;
2. Feasibility: screening-to-recruitment ratio < 2 and recruitment of ≥ 1 participant/site/week;
3. Safety: adverse events and serious adverse events;
4. Protocol compliance: ≥ 80% protocol compliance and exercise intervention commenced at two post-operative weeks;
5. ADAS-cog: difference of at least 2 on the ADAS-cog scale; and
6. Muscular strength: ≥ 15% improvement in strength.

The analysis will be on an intention-to-treat basis. Data collected for feasibility and safety will be analysed using descriptive statistics (mean ± standard deviation) to explore possible effects of the intervention. Mean changes in outcomes over time will be summarised graphically and descriptively within each group, with odds ratio and confidence intervals. As this is a pilot study, it is not powered to detect clinically meaningful effects. Feasibility data will be analysed to determine whether it is worth conducting a large-scale trial. A statistician not involved in the study and the student researcher will assess these parameters at the end of the trial.

### Data monitoring, harm and auditing

The team has nominated Professor Alistair Royse to monitor all aspects of this project. As this is a small randomised trial without any interim analysis, there will not be a separate Data and Safety Monitoring Board. Any adverse outcomes will be reported via the CI Prof Colin Royse to the Melbourne Health HREC.

If a patient suffers any injuries or complications as a result of this research project, they will be advised to contact the study team as soon as possible and will be assisted with arranging appropriate medical treatment. If they are eligible for Medicare, they can receive any medical treatment required to treat the injury or complication, free of charge, as a public patient in any Australian public hospital.

In accordance with Melbourne Health HREC, details of patient enrolment, adverse events, withdrawal and study completion will be recorded in their electronic medical records. Annual reports will be submitted to facilitate their auditing and monitoring of registered trials.

## Discussion

The benefits of cardiac rehabilitation in optimising post-operative physical and functional recovery are well recognised globally. Exercise has also been shown to improve cognitive function. However, cognitive function is not assessed in the cardiac population pre-operatively, nor monitored post-operatively. Thus, the impact of cardiac rehabilitation on post-operative cognitive recovery is unknown.

Cardiac rehabilitation traditionally involves low-intensity aerobic exercise and at times low resistance weights [9]. Balachandran et al. [33] have shown that coughing produced the greatest sternal micromotion and pain when compared to dynamic upper limb and trunk tasks [33], whilst Swanson and LaPier have shown that common daily activity, such as moving from side-lying to sitting, opening doors and pulling out a chair require the movement of loads greater than that permitted by current sternal precautions [50]. However, despite evidence that demonstrates the safety of upper limb exercise in this population [33], resistance training is not typically commenced until 12 weeks post-operatively if at all, as current sternal precautions restrict the use of the upper limbs and trunk for 8–12 weeks [18, 51, 52]. As such, cardiac rehabilitation programs have been reported to adopt a conservative and cautionary approach to the prescription of upper limb exercises and in particular to resistance training of the upper limb. This inadvertently encourages patients to become physically inactive, potentially delaying their recovery and contributing to deterioration of physical and cognitive function. There is emerging evidence to support the safety and efficacy of moderate-intensity exercise, and upper limb and thoracic progressive exercise. Resistance training accelerates improvements in cardiovascular fitness and muscle strength and reduces inflammation, cognitive dysfunction and sarcopenia, which can persist for several months after surgery [11, 38, 53–59]. However, the effects of early moderate-intensity resistance training following cardiac surgery are unknown.

Cognitive impairment is a frequent complication of cardiac surgery that is not typically addressed in cardiac rehabilitation programs. There is evidence associating resistance-based exercise programs with improved cognitive outcomes in older adults (aged 65 years and older) [38, 60] and adults with mild cognitive impairment [41, 61], providing a strong rationale for incorporating resistance training into rehabilitation training after surgery. To our knowledge, this pilot study is the first trial to comprehensively assess the safety and feasibility, cognitive recovery, physical and functional recovery and participant perceived impact of early resistance training as a form of cardiac rehabilitation. The results of this pilot study will be used to determine the feasibility of a future large-scale randomised controlled trial that promotes the integration of early resistance training into existing aerobic-based cardiac rehabilitation programs in Australia.

### Trial status

Version 5, 17 December 2019

Recruitment commenced on 16 April 2018 and concluded on 31 August 2019.

## Supplementary information

**Additional file 1.** SPIRIT 2013 Checklist: Recommended items to address in a clinical trial protocol and related documents

**Additional file 2.** Resistance Training Program Exercises (Weeks 1-6)

**Additional file 3.** Resistance Training Program Exercises (Weeks 7-12)

**Additional file 4.** Sternal ultrasound protocol

**Additional file 5.** Master Patient Information Consent Form (PICF)

## Data Availability

Requests for sharing of the trial protocol, full study report and anonymised participant-level dataset beyond that of the information detailed in this protocol will be considered on a case-by-case basis and made at the discretion of the research team.

## Abbreviations

MACCE: Major adverse cardiac and cerebral events
SIS: Sternal Instability Scale
RPE: Rating of Perceived Exertion
ADAS-cog: Alzheimer’s Disease Assessment Scale and cognitive subscale
MMSE: Mini Mental State Examination
IADL: Instrumental Activities of Daily Living
PostopQRS: Postoperative Quality of Recovery Scale
FDQ: Functional Difficulties Questionnaire
FSST: Four Square Step Test
BMI: Body mass index
FVC: Forced vital capacity
FEV1: Forced expiratory volume in 1 s
US: Ultrasound
