# Supplementary material for: Effects of Supervised Early Resistance Training versus standard care on cognitive recovery following cardiac surgery via median sternotomy (the SEcReT study): protocol for a randomised controlled pilot study
Source: Trials. 2020 Jul 15;21:649. doi: 10.1186/s13063-020-04558-x (PMC7362413; doi:10.1186/s13063-020-04558-x)
Supplement: Supplementary file 2 — Additional file 2. Resistance Training Program Exercises (Weeks 1-6) [file 13063_2020_4558_MOESM2_ESM.docx]

**Appendix A: Resistance Training Program Exercises (Weeks 1-6)**

**
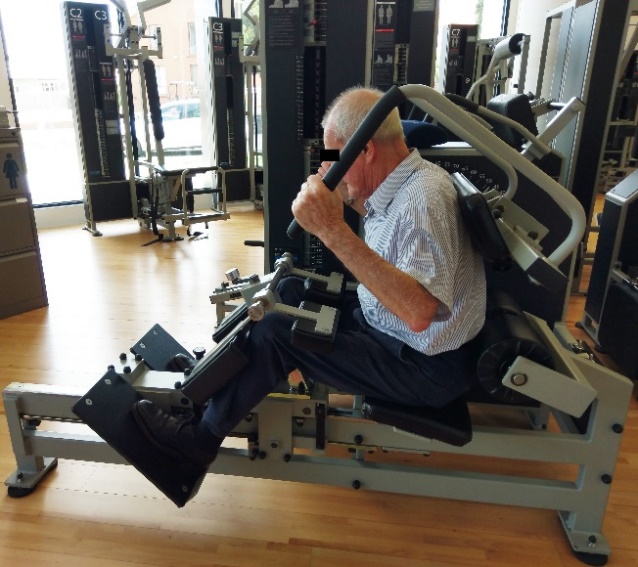

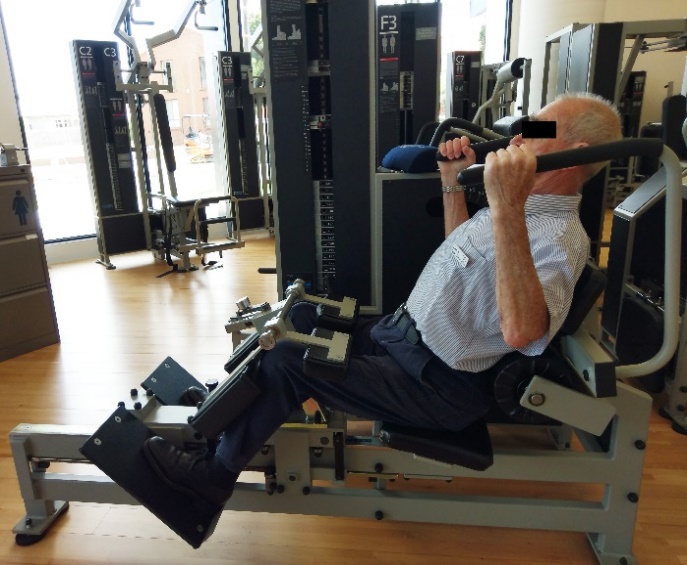
**

**A)**

**B)**

1. Back Extension *A) starting position B) end position*

**
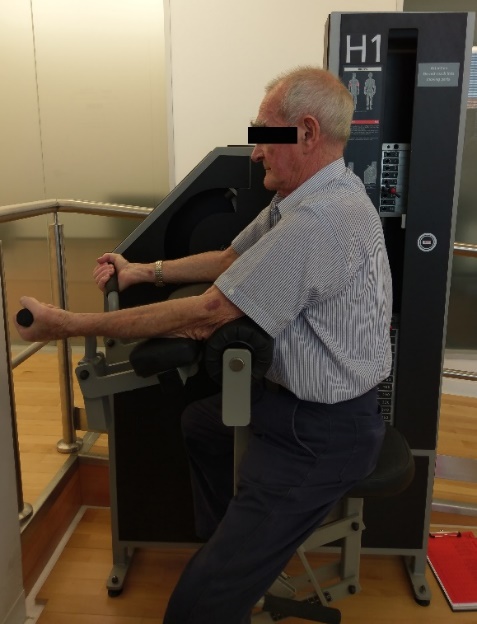

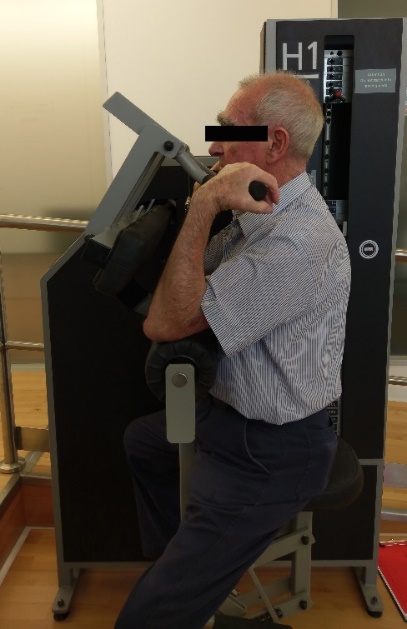
**

**B)**

**A)**

1. Biceps curl *A) starting position B) end position*

**
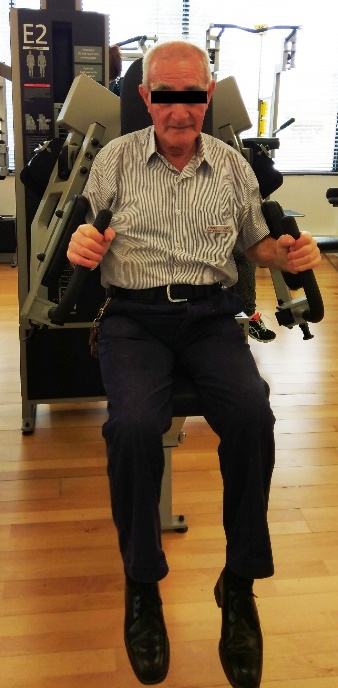

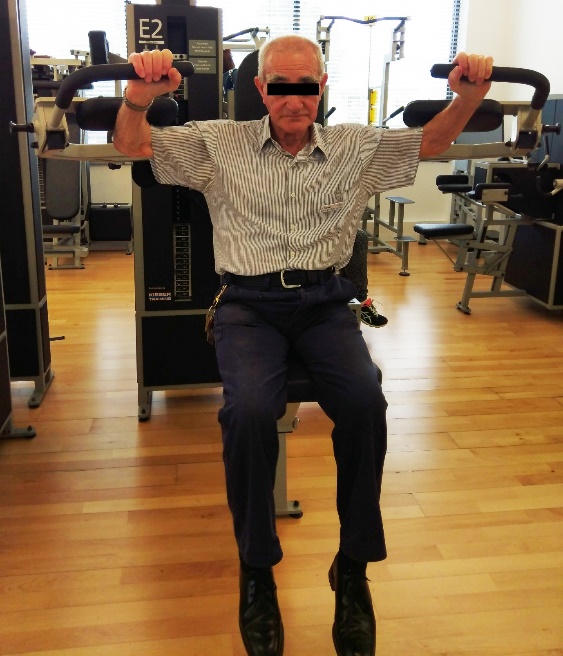
**

**B)**

**A)**

1. Lateral Raise *A) starting position B) end position*

**
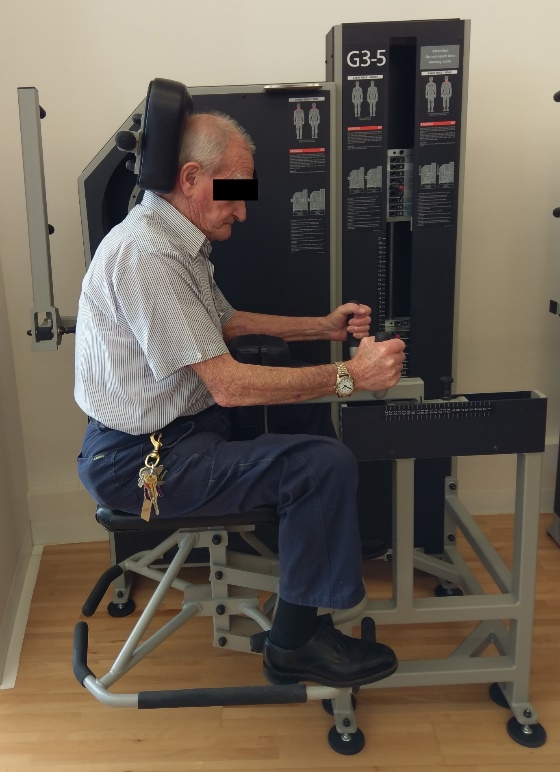

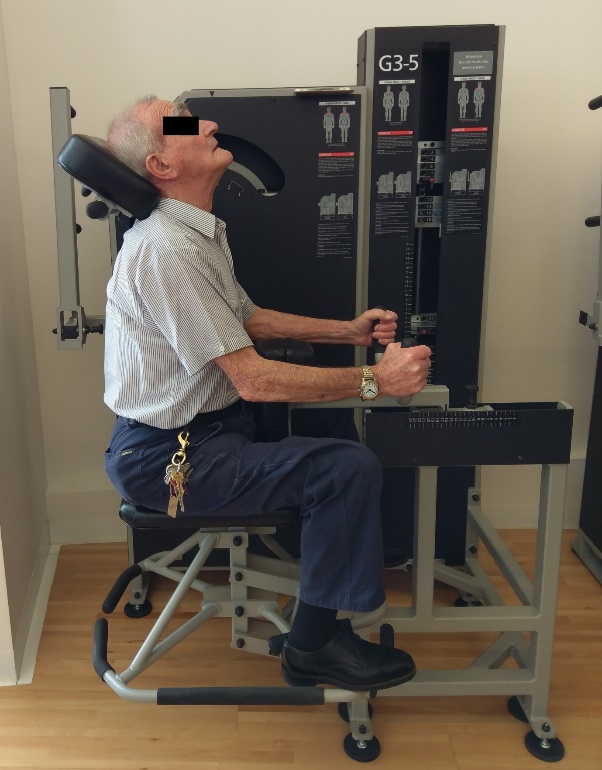
**

**B)**

**A)**

1. Neck Extension (added from week 2) *A) starting position B) end position*

**
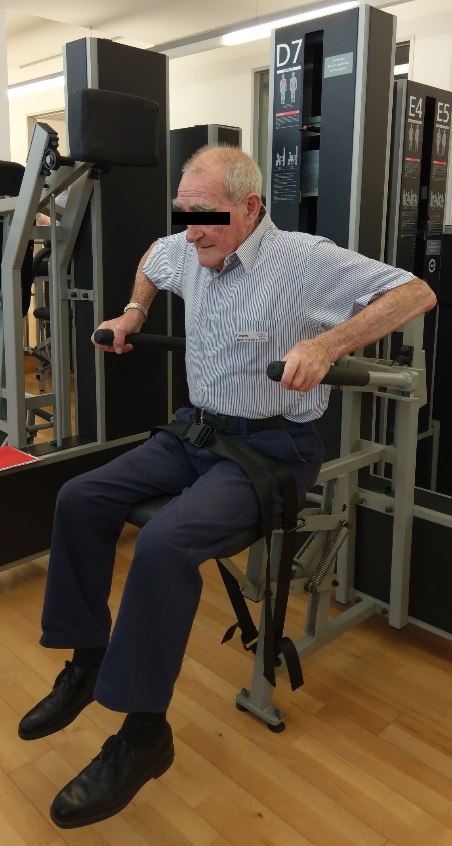

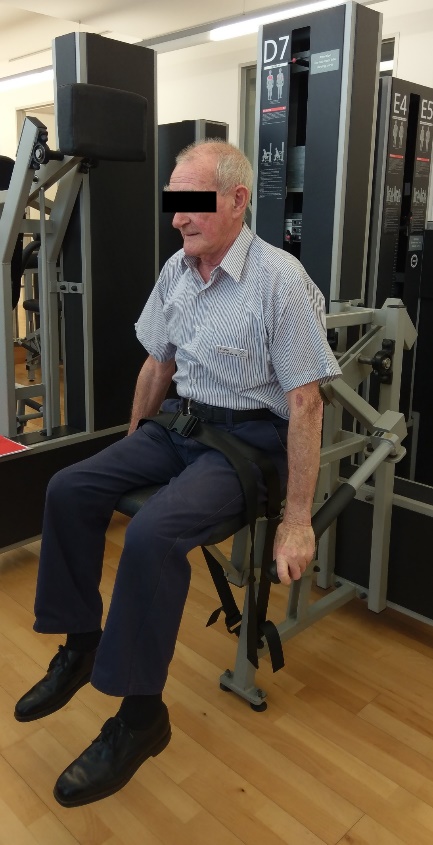
**

**A)**

**B)**

1. Triceps pushdown *A) starting position B) end position*

**
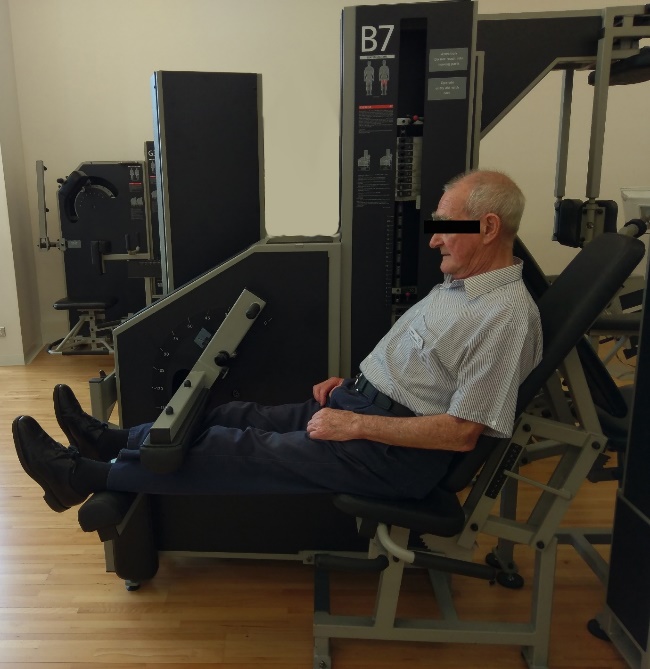

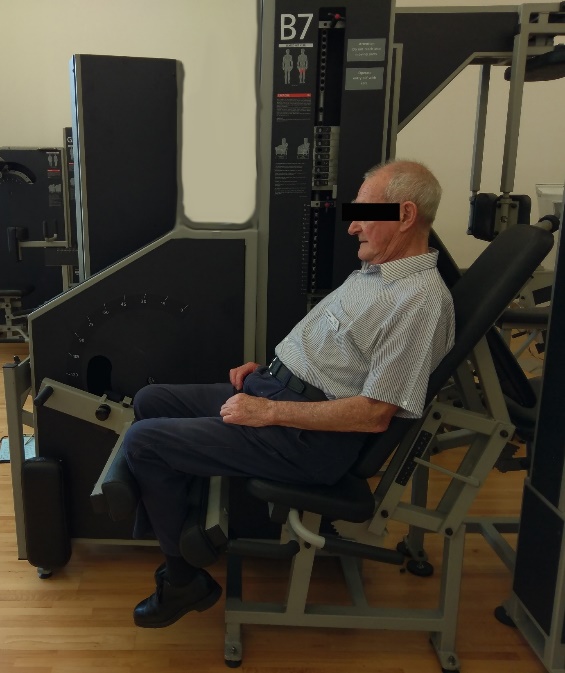
**

**B)**

**A)**

1. Knee flexion (Seated hamstring curl) *A) starting position B) end position*

**
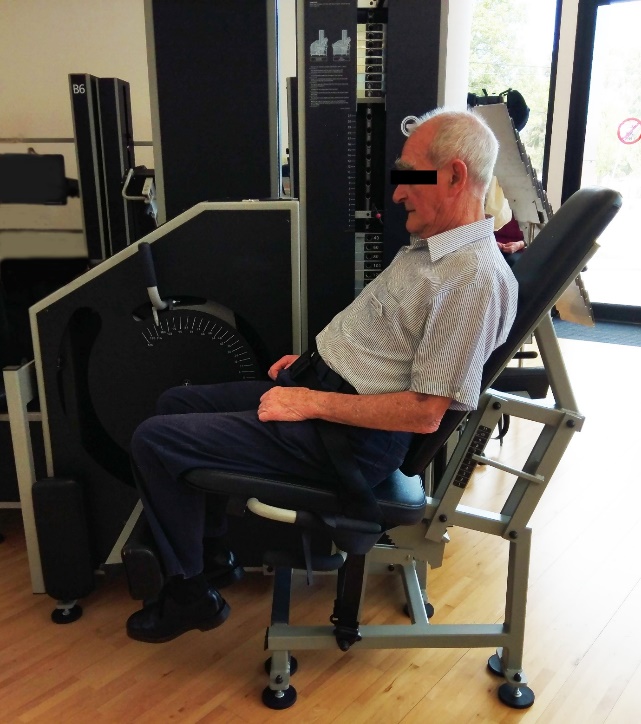

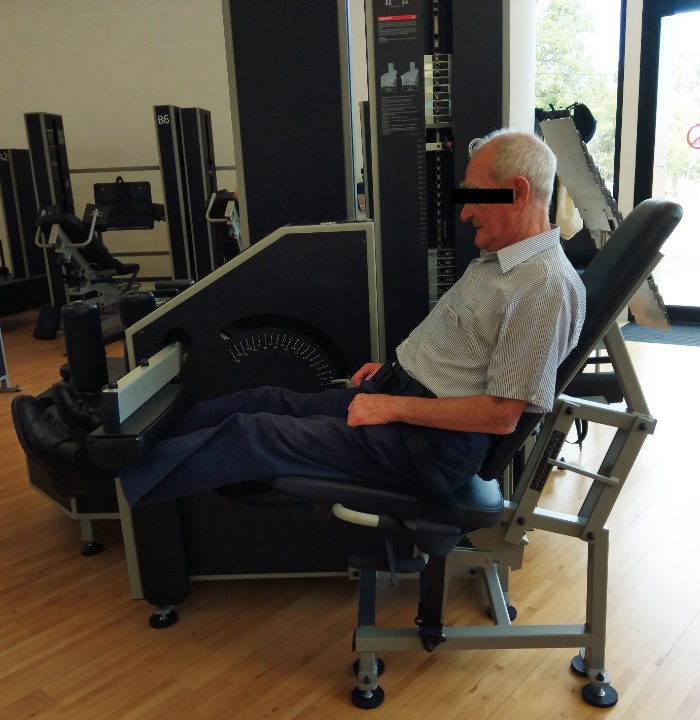
**

**B)**

**A)**

1. Knee Extension *A) starting position B) end position*
