# Supplementary material for: Effects of Supervised Early Resistance Training versus standard care on cognitive recovery following cardiac surgery via median sternotomy (the SEcReT study): protocol for a randomised controlled pilot study
Source: Trials. 2020 Jul 15;21:649. doi: 10.1186/s13063-020-04558-x (PMC7362413; doi:10.1186/s13063-020-04558-x)
Supplement: Supplementary file 3 — Additional file 3. Resistance Training Program Exercises (Weeks 7-12) [file 13063_2020_4558_MOESM3_ESM.docx]

**Appendix B: Resistance Training Program Exercises (Weeks 7-12)**

**
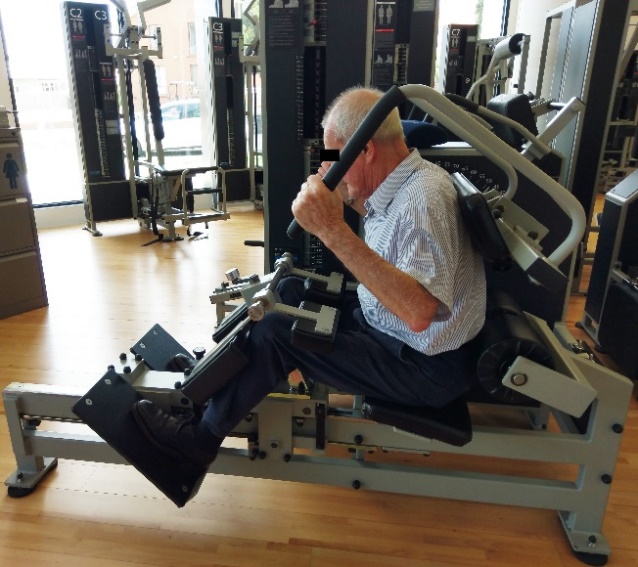

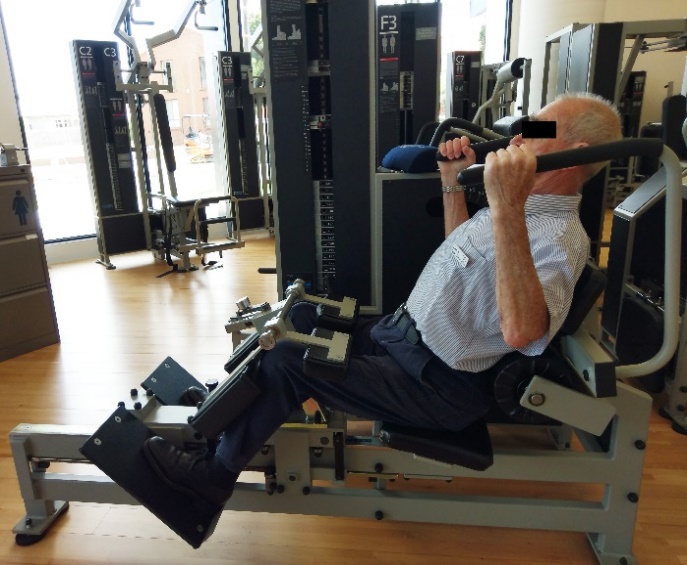
**

**A)**

**B)**

1. Back Extension *A) starting position B) end position*

**
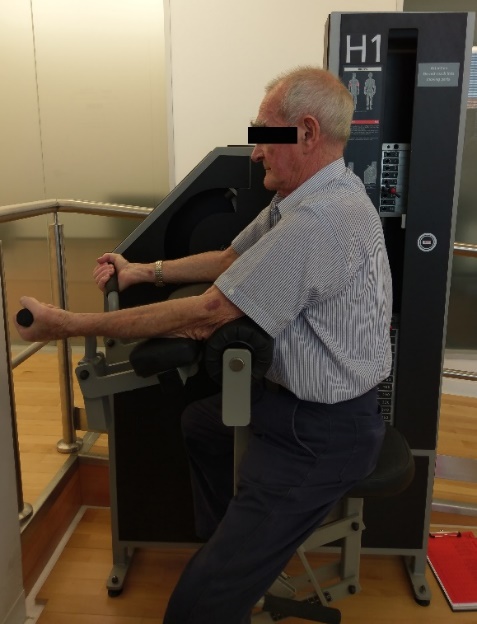

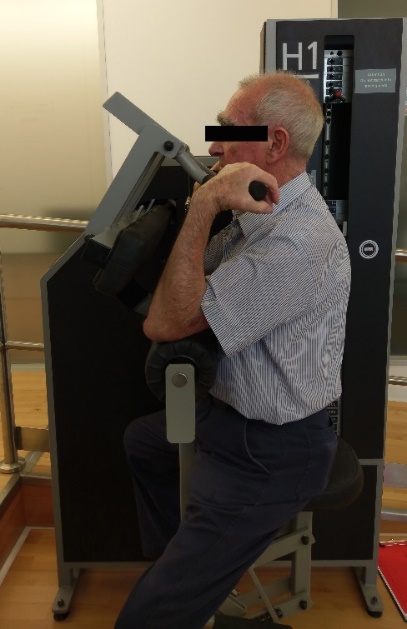
**

**B)**

**A)**

1. Biceps curl *A) starting position B) end position*

**
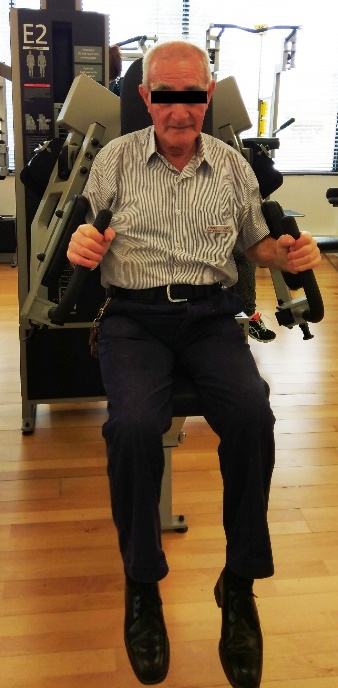

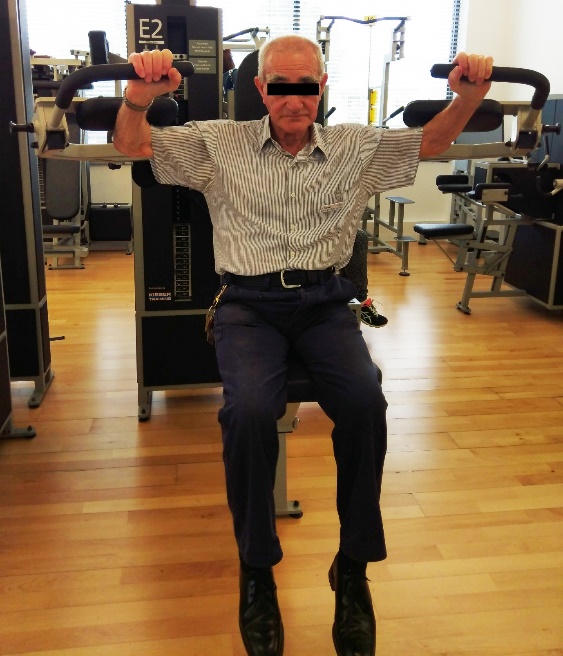
**

**B)**

**A)**

1. Lateral Raise *A) starting position B) end position*

**
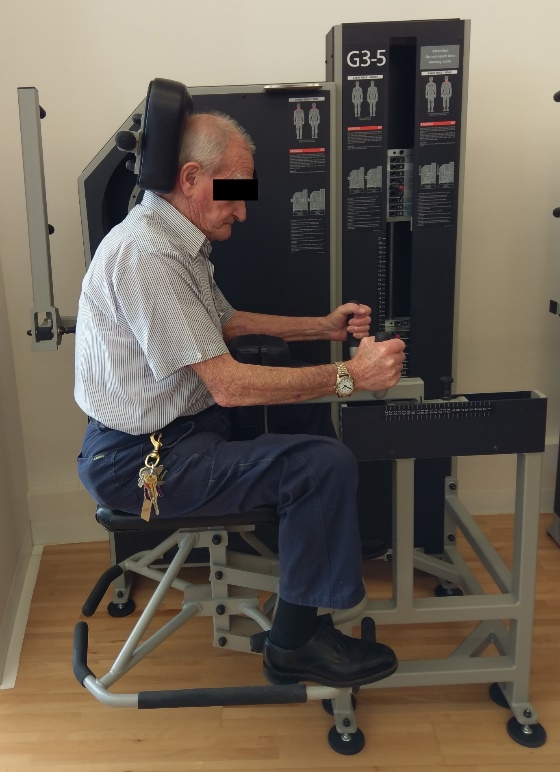

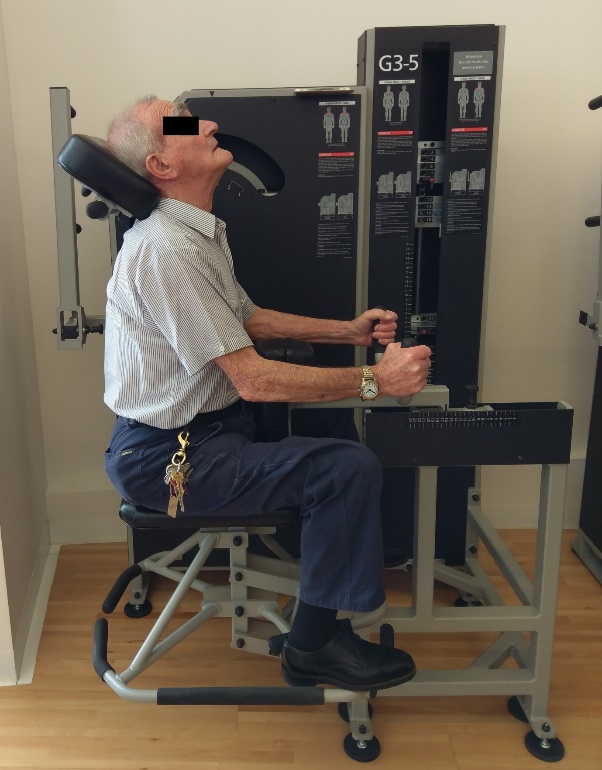
**

**B)**

**A)**

1. Neck Extension *A) starting position B) end position*

**
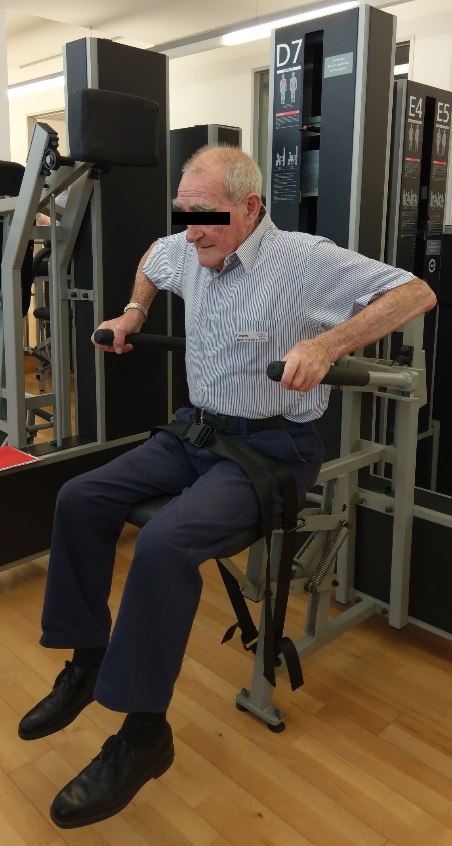

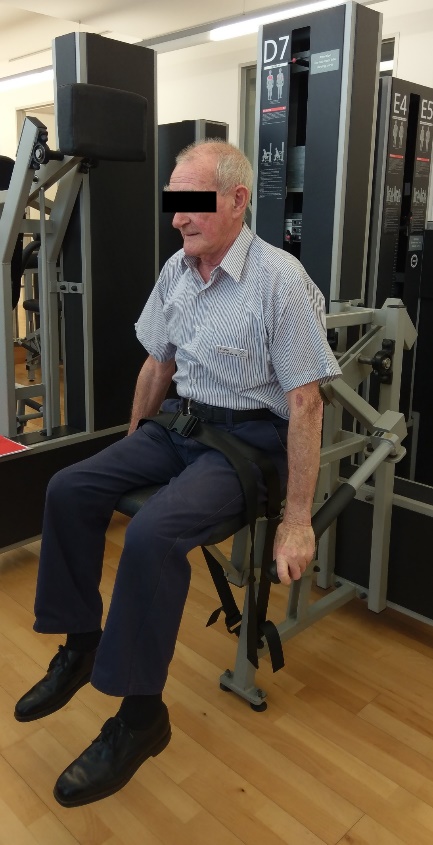
**

**A)**

**B)**

1. Triceps pushdown *A) starting position B) end position*


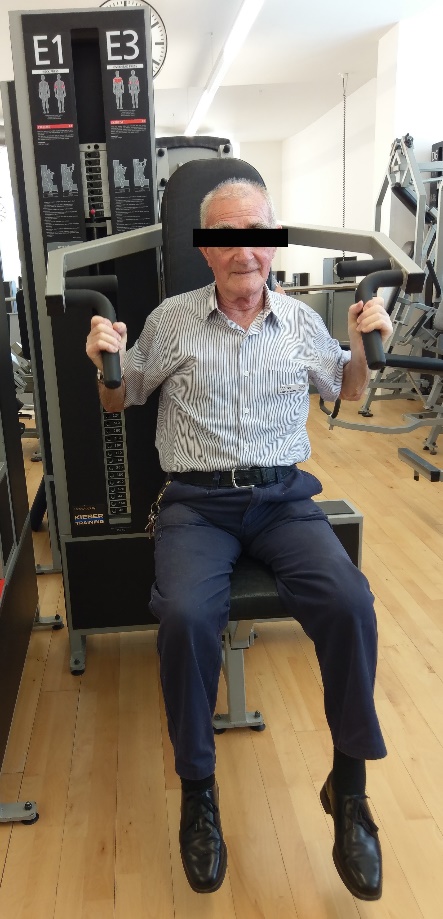

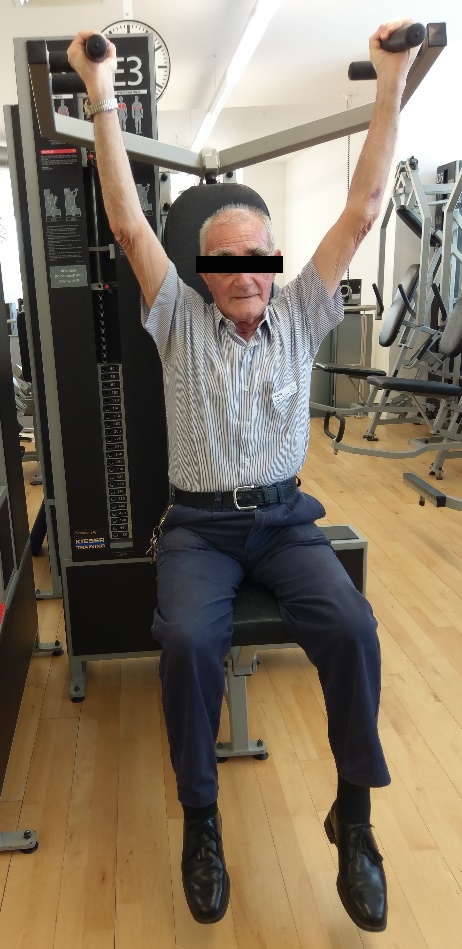


**A)**

**B)**

1. Shoulder press *A) starting position B) end position*


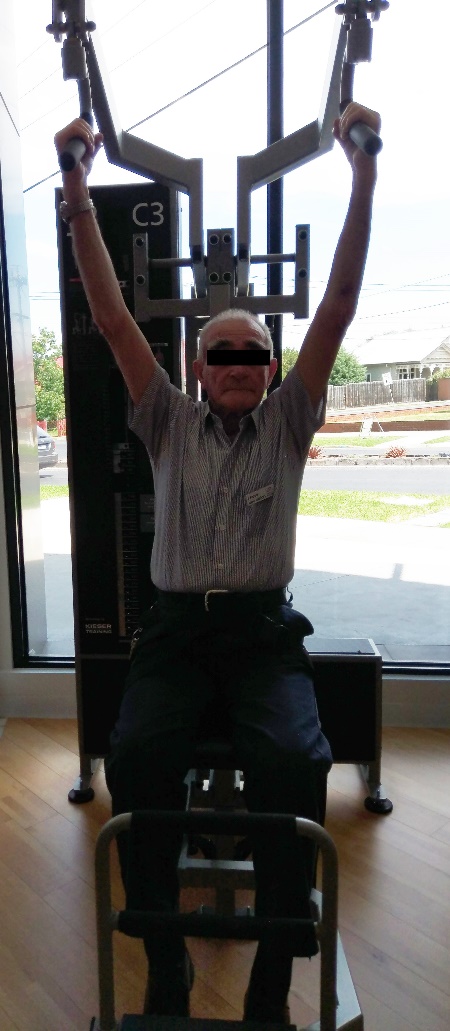

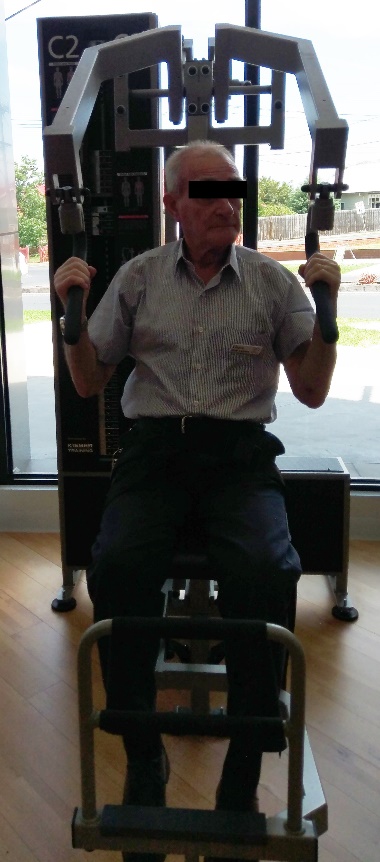


**A)**

**B)**

1. Latissimus pulldown *A) starting position B) end position*


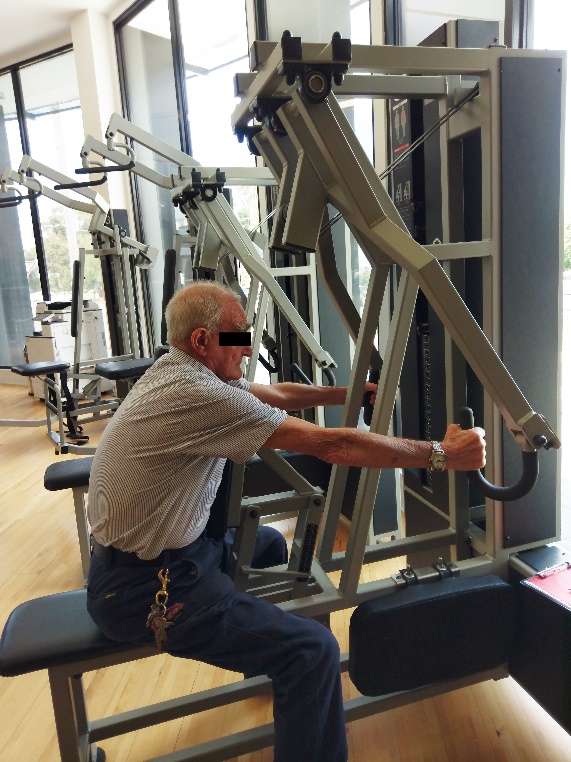

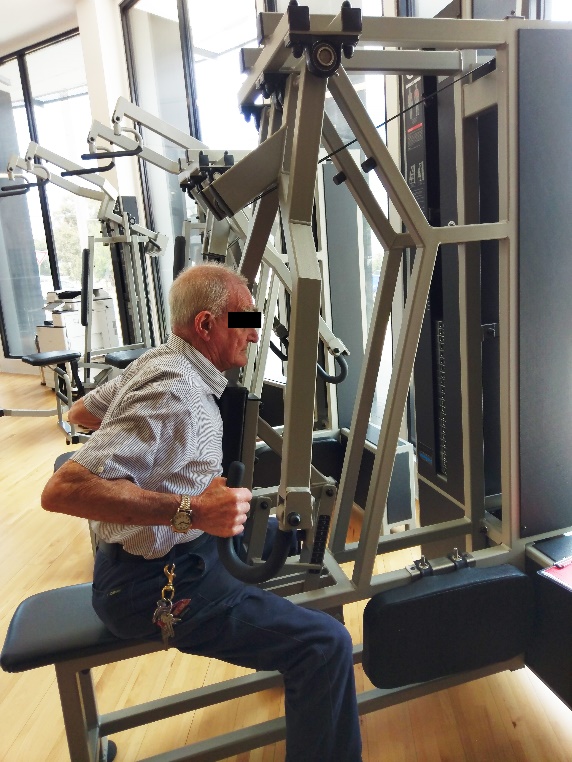


**B)**

**A)**

1. Seat row *A) starting position B) end position*


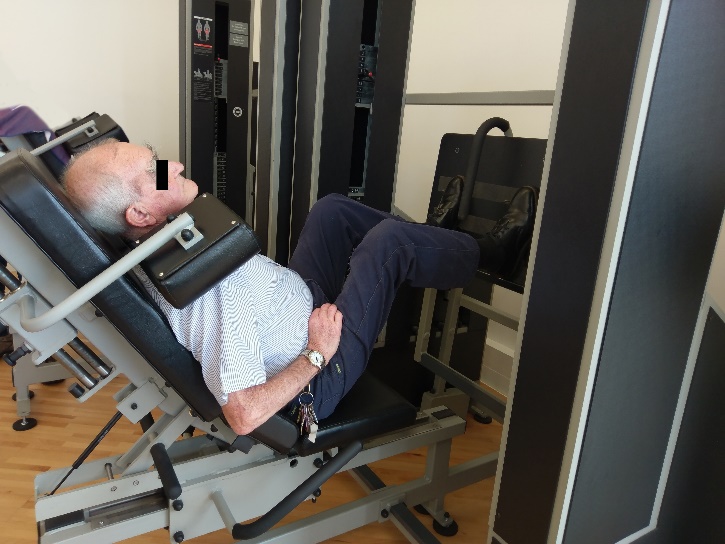

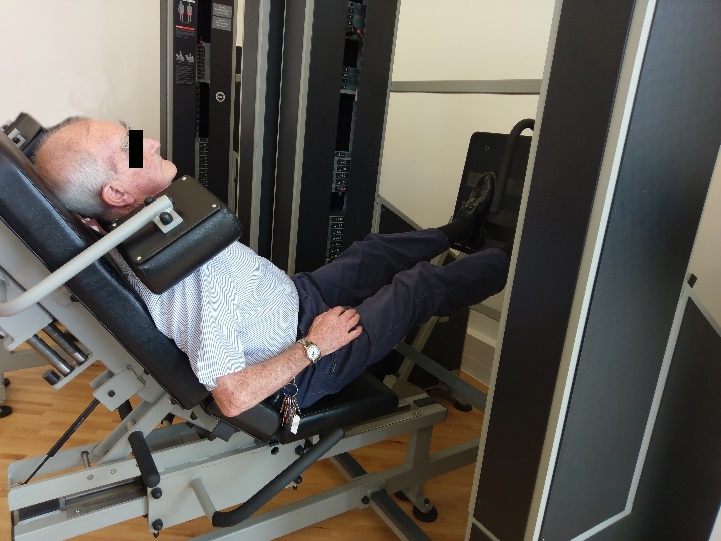


**B)**

**A)**

1. Leg press *A) starting position B) end position*


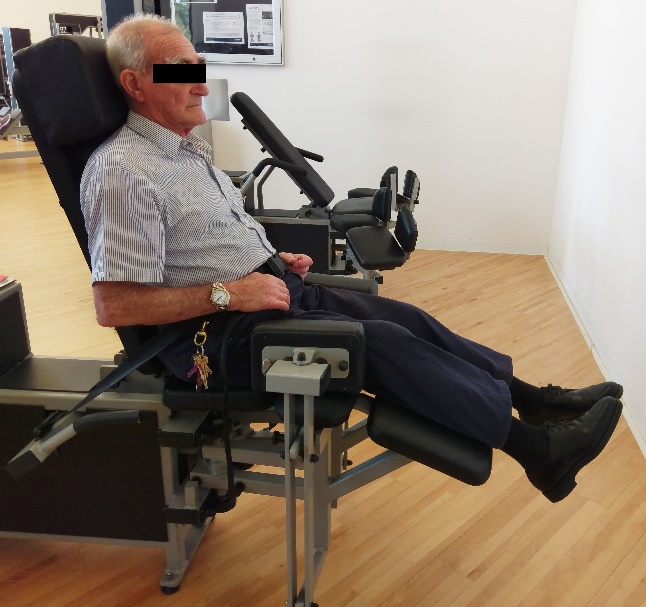

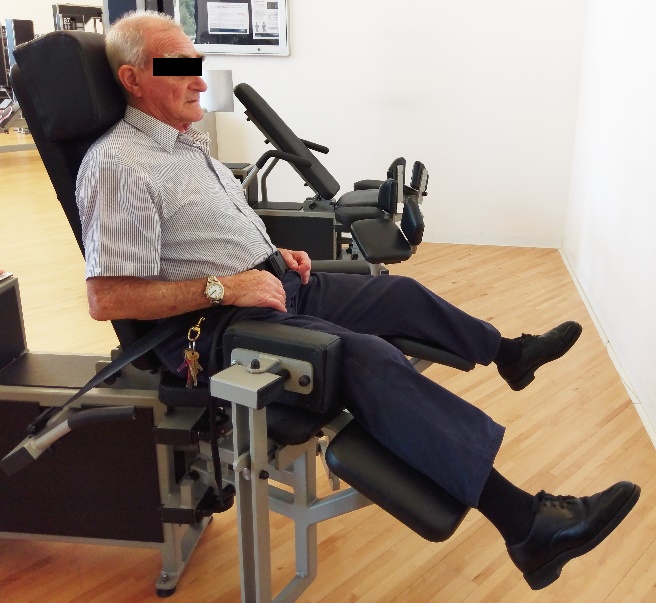


**B)**

**A)**

1. Hip abduction *A) starting position B) end position*


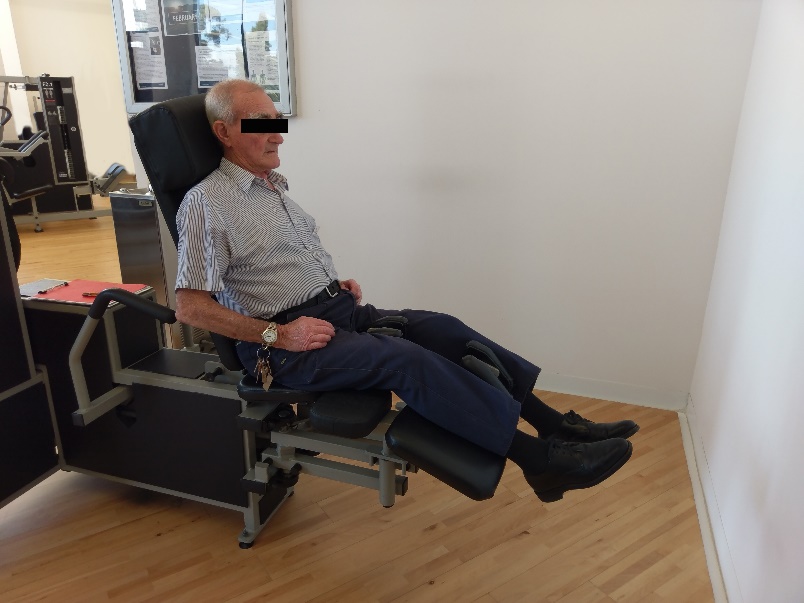

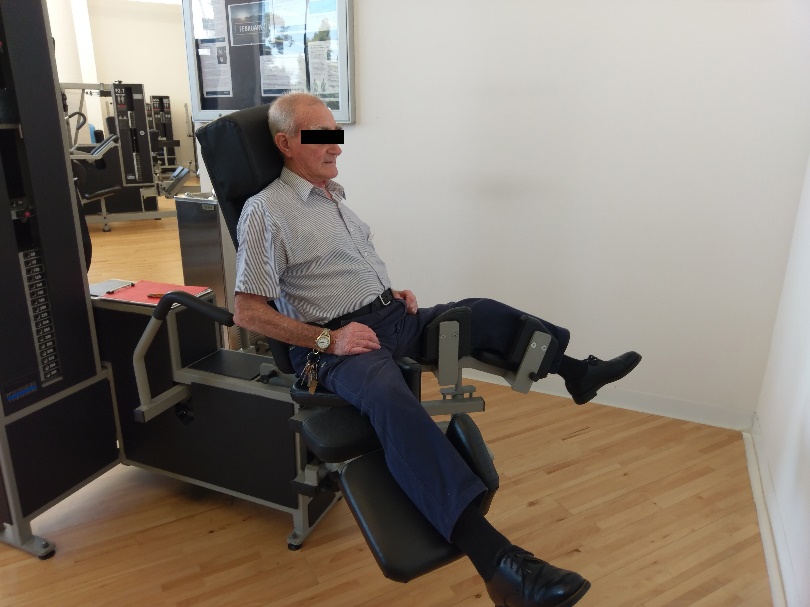


**B)**

**A)**

1. Hip adduction *A) starting position B) end position*
