# Supplementary material for: Effects of Supervised Early Resistance Training versus standard care on cognitive recovery following cardiac surgery via median sternotomy (the SEcReT study): protocol for a randomised controlled pilot study
Source: Trials. 2020 Jul 15;21:649. doi: 10.1186/s13063-020-04558-x (PMC7362413; doi:10.1186/s13063-020-04558-x)
Supplement: Supplementary file 4 — Additional file 4. Sternal ultrasound protocol [file 13063_2020_4558_MOESM4_ESM.docx]

**Appendix C: Sternal ultrasound protocol**
